# Is “Trigonum sacrale” a real equilateral triangle? Anatomic consideration of sacral hiatus in adult: A three-dimensional CT study for reliable caudal access

**DOI:** 10.1097/MD.0000000000032098

**Published:** 2022-11-25

**Authors:** Norishige Kuramitsu, Satoshi Nozawa, Yasumichi Sakaguchi, Kazunari Yamada, Chizuo Iwai, Haruhiko Akiyama

**Affiliations:** a Department of Orthopedic Surgery, Gifu University School of Medicine, Gifu, Japan; b Department of Orthopedic Surgery, Mino Municipal Hospital, Mino, Japan.

**Keywords:** anesthesia, caudal, imaging, sacral hiatus, sacrum, three-dimensional

## Abstract

This study is the first attempt to examine anatomical characteristics using three-dimensional computed tomography (3DCT) bone images with some parameters, in order to achieve correct and uncomplicated accesses. In addition, the study addresses a long-standing problem in the field and evaluates whether the trigonum sacrale forms an equilateral triangle or not. A detailed anatomic study of the sacral region was carried out on 91 patient 3DCT images. The CT data, in DICOM format, was read into VINCENT software from Fuji Film, with a slice thickness of 0.5 mm. The average length of sacral hiatus was 28.6 ± 8.4 (range 13.8–45.2 mm). The average width of sacral hiatus at the level of sacral cornua was 10.9 ± 2.7 (range 3.8–16.5 mm). The ratio between the length of the oblique and base line formed by the sacral triangle was 0.81 ± 0.12 (range 0.54–1.00). Using 3DCT images translated by the volume rendering technique, we can remove soft tissue from bones virtually. A slice thickness of 0.5 mm makes it a fine image, and permits meticulous measurement, which is different from previous cadaveric studies. Interestingly, our data showed that the ratio between oblique and base line on sacral triangle was <1.0, average 0.81. Findings demonstrated that the trigonum sacrale is not an equilateral triangle. This is useful information for the identification of the sacral hiatus when the landmark-based technique is employed.

## 1. Introduction

The caudal epidural block (CEB) has been used for many years, and is the easiest and safest approach for patients with leg pain. The needle insertion method using anatomical landmarks is a simple and easily procedure which does not require special equipment. Conversely, a failure rate of 25% has been reported in previous papers.^[1–4]^ Previous studies reported that the apex of the sacral hiatus and the superolateral sacral crests (Trigonum sacrale) form an equilateral triangle, constituting common indicators of anatomical landmarks.^[5–9]^ However, most of the previous reports have focused on measurements based on the use of the dry sacral bone without the ilium. To the best of our knowledge, there are no reports on the evaluation of trigonum sacrale using the landmark of the posterior superior iliac spine (PSIS) to the sacral hiatus. The knowledge of sacral hiatus anatomy is essential in clinical situations requiring CEB for diagnostic and therapeutic procedures. In fact, elucidating whether trigonum sacrale is equilateral or not could decrease the risk of pricking incorrect part.

To our knowledge, this is the first study that has examined the positional relationship between the PSIS to the sacral hiatus based on three-dimensional computed tomography (3DCT) images using medical image analysis software for the measurements of the morphological characteristics. We also evaluated whether the trigonum sacrale forms an equilateral triangle or not.

## 2. Methods

**Figure 1.**
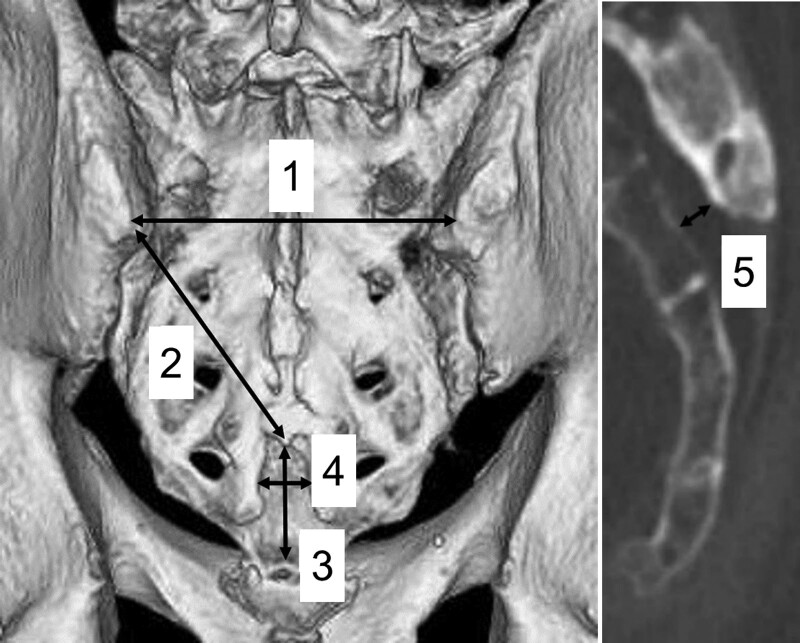
Measured parameters. Length between right and left posterior superior iliac spines (PSISs), distance from sacral hiatus apex to the left side of PSIS, height of sacral hiatus, width of the sacral hiatus at the level of sacral cornua, and depth of sacral hiatus at the level of its apex.

A detailed anatomic study of the sacral region was conducted based on 3DCT images, which included 50 males and 41 females, with the mean age of 71.4 years (range: 11–98 years). Patients distribution is, 6 cases (6.5%) in 10 to 49 year-old group, 11 cases (12%) in 50 to 59 year-old, 18 cases (19.8%) in 60 to 69 year-old, 20 cases (22%) in 70 to 79 year-old, 32 cases (35.2%) in 80 to 89 year-old and 4 cases (4.4%) in 90 to 99 year-old. The abdominopelvic CT images of 91 patients taken for screening at the general surgery department in Mino city hospital were used in this study. This observational study is approved by Mino city hospital IRB (institutional review board) as #2014024 using opt-out consent. The CT data were saved in a digital image communications in medicine format, and were read in the software VINCENT from Fuji Film with a slice thickness of 0.5 mm. Anatomical measurements were performed on 3DCT images with the volume rendering technique by an orthopedic surgeon. First of all, we evaluated the presence of the sacral hiatus and sacral cornua landmarks. Subsequently, the length between right and left PSISs, distance from sacral hiatus apex to the left of PSIS, the height of sacral hiatus, the width of the sacral hiatus at the level of sacral cornua, and the depth of sacral hiatus at the level of its apex were measured (Fig. 1). Further, we examined whether an equilateral triangle can be drawn to connect the PSISs and the sacral hiatus, as described in previous publications. We also assessed ratio of the oblique and baseline sides formed by the trigonum sacrale for its number, differences between males and females, and comparison between ages.

### 2.1. Statistical analysis

Statistical analysis was performed using SPSS (version 25, IBM Corporation, Armonk, NY) and the results were expressed as mean ± standard deviation (SD). The Mann–Whitney test or one-way Analysis of Variance (ANOVA) using Turkey range test were used for comparisons between pairs of groups. This sample size allowed us to detect an effect size of f = 0.80 based on a power (1 − β) of 70%, an α error of 0.05 in one-way ANOVA test, calculated using Gpower software. *P* values < .05 were considered significant in all analyses.

### 2.2. Power estimation

Power analysis was conducted to determine the appropriate sample size for this study in comparison between males and females. Sample size was calculated based on the target power (0.70) and alpha-level (α = 0.05) using Gpower computer software. The power estimation suggested a minimum sample size 41.

## 3. Results

Agenesis of the sacral hiatus was found in eleven (12.1%) sacra. The average distance between PSISs was 80.2 ± 10.0 (range 58.2–109.2 mm). The distance between left PSIS and sacral apex was 64.1 ± 8.8 (range 45.6–96.7 mm). The average height of sacral hiatus was 28.6 ± 8.4 (range 13.8–45.2 mm). The average width of sacral hiatus at the level of sacral cornua was 10.9 ± 2.7 (range 3.8–16.5 mm) Narrowing of the hiatus (values less than mean - SD value) was detected in ten (10.9%) sacra. The average depth of the sacral hiatus at the level of its apex was 6.3 ± 1.6 (range 1.6–9.5 mm) (Table. 1).

**Figure 2.**
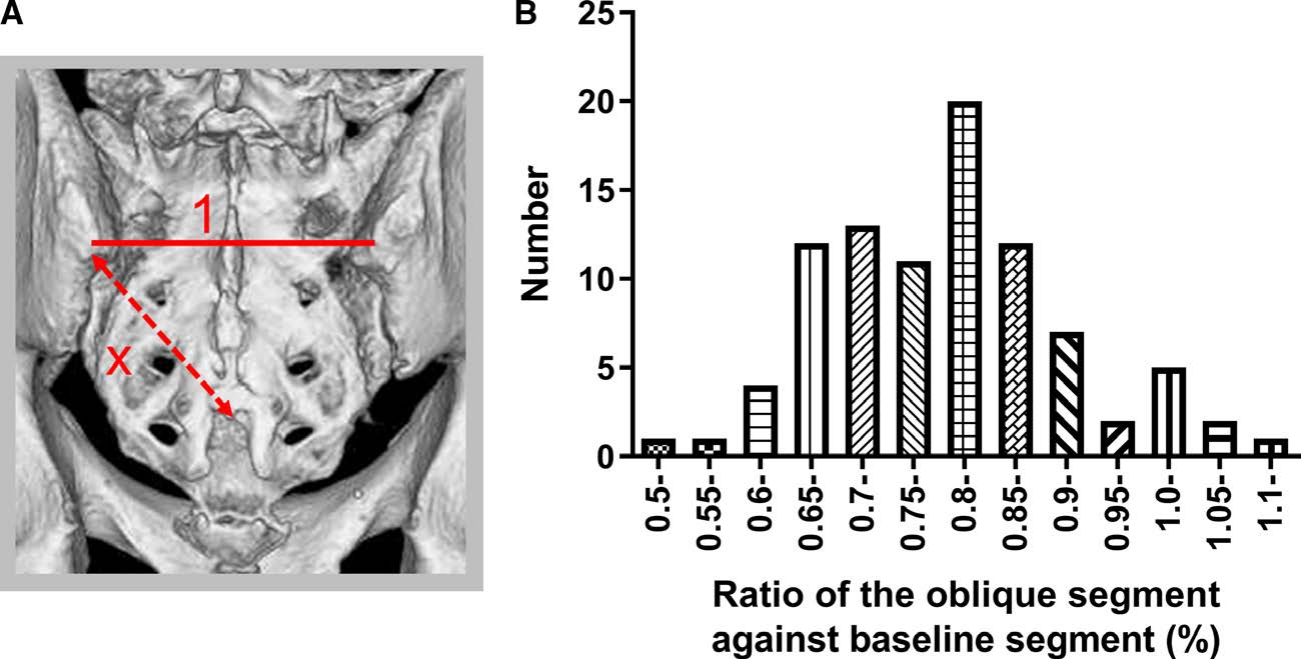
(a) X means ratio of the oblique segment against baseline segment in case that baseline length is 1. (b) The number of each ratio (X) group are shown. The mean ratio between the length of the oblique and baseline formed by the trigonum sacrale was 0.81.

**Figure 3.**
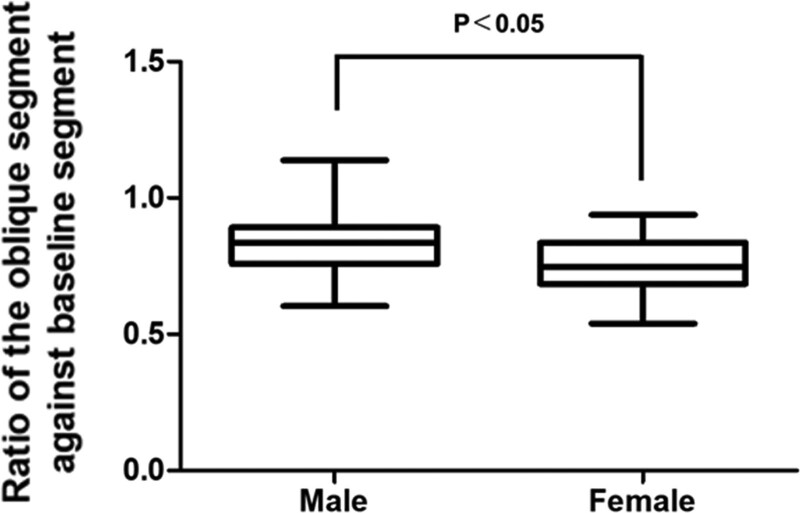
Comparison of the ratios of the oblique and baseline on the trigonum sacrale between males and females.

**Figure 4.**
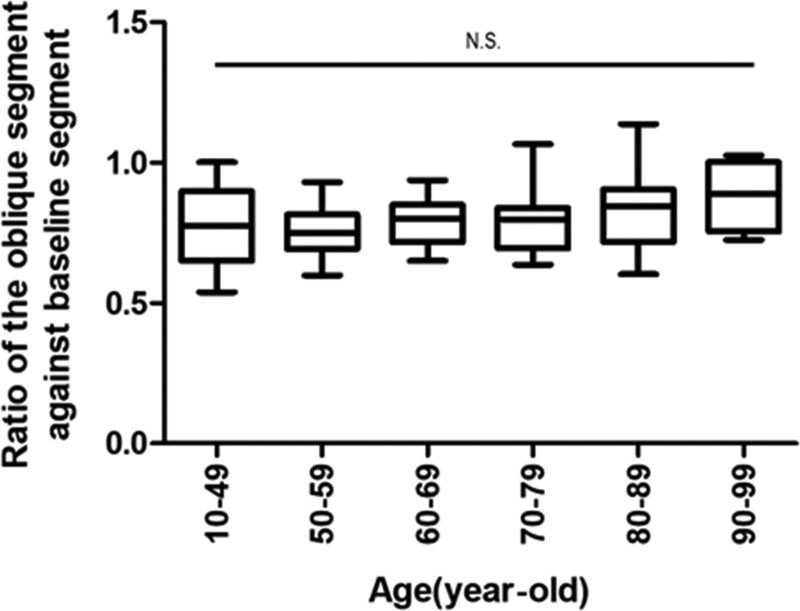
Comparisons of the ratio of the oblique segment against baseline on the trigonum sacrale between ages are shown. There is no statistical difference between the groups (one way ANOVA test).

The ratio between the length of the oblique and baseline formed by the trigonum sacrale was 0.81 ± 0.12 (range 0.54–1.00) (Fig. 2). The ratio was significantly larger in males than in females, 0.83 ± 0.12, 0.77 ± 0.10, respectively (*P* < .05) (Fig. 3). There were not any obvious differences between the ages (*P* > .05) (Fig. 4).

## 4. Discussion

This study is the first report to measure the length between PSIS to the sacral hiatus using 3DCT with the ilium connected to the sacrum. By using the software VINCENT, the distance between two points on 3DCT could be measured accurately, which is difficult using only the sacrum.

In CEB, methods such as the landmark-based technique, fluoroscopy- and ultrasound-guided techniques are generally used. The landmark-based technique using anatomical landmarks is generally the most used because it is a simple and easy procedure and requires no special equipment.^[1–6]^ In children, the success rate of the landmark-based technique was over 96%, while in adults it was only 68% to 75%.^[1,5,7–9]^ One of the factors associated with the higher failure rate in adults is the wide anatomic variation. Therefore, a detailed evaluation of anatomical landmarks is useful for clinicians. Another landmark suggested in various reports is the equilateral triangle structure formed by the lines of the right and left lateral sacral crest and the apex of the sacral hiatus on dried sacral bone.^[6,10-12]^ The PSISs lie on the lateral sacral crest at the level of the first sacral foramen. Senoglu et al^[6]^ reported that trigonum sacrale formed an equilateral triangle, while Bagheri et al^[10]^ reported that in 55% cases, both sides of the triangle were smaller than the base of the triangle. Knowledge on whether the trigonum sacrale from PSIP is an equilateral triangle or not constitutes useful data, especially in obese patients who cannot be easily palpated owing to the thickening of their soft tissues. However, in past reports, the measurements were conducted by superolateral sacral crests, and not by PSISs. This is probably because it is difficult to maintain strict sacral and iliac bone continuity when processing dry sacral bone.

In our study, 3DCT images were used. These were translated by the volume rendering technique. In this way, we could remove the soft tissue from bones virtually. A slice thickness equal to 0.5 mm makes its fine image and permits meticulous measurement, which is different from previous cadaveric studies. Interestingly, our data showed that the ratio between oblique and baseline on the trigonum sacrale from PSIS was <1.0 (average value: 0.81). Previously, the trigonum sacrale was commonly thought to exhibit an equilateral triangle, but this study demonstrated that this assertion was incorrect. The mean ratio between the hypotenuse and base of the isosceles triangle was 0.81 (Fig. 2), and the female group had a smaller hypotenuse ratio compared with the male group (Fig. 3).

The lower values in the previous report were attributed to the superior lateral sacral crest which was slightly medial to PSISs. These findings may be related to the larger baseline measured using PSISs. The left border of the triangle was similar to the previous reports (previously reported average range: 60.0–75.0, value in our report: 64.1), but the base of triangle was the largest measure we reported (previously reported average 60.0–75.5, value in our report: 80.2).^[2,6,10-16]^

To assess the accuracy of the 3DCT, we compared our data with past reports (Table 1).^[2,6,10-16]^ The height of the sacral hiatus in our study was 28.6 ± 8.4 (range 13.8–45.2 mm), thus exhibiting a broad variation as reported previously. If the cornua is absent or if the patient is obese, in addition to a shorter hiatus, it will be difficult to insert the needle into the caudal epidural space accurately. In contrast, higher hiatus facilitates needle insertion in the sacral canal but concurrently increases the risk of dural sac damage. Bagheri et al^[10]^ reported that the height of the sacral hiatus ranged from 21 to 40 mm in 80% of the specimens. This height range was also found in 78% of the specimens in our study.

**Table 1:** Comparison of measurements between different studies [mean ± SD(range)].

Parameters (mm)	Present Study (2022)	Bagheri et al^[10]^(2017)	Nadeem et al^[11]^ (2018)	Mustafa et al^[12]^ (2012)	Aggarwal et al^[13]^ (2009)	Kumar et al^[14]^ (2009)	Senoglu et al^[6]^ (2005)	Sekiguchi et al^[15]^ (2005)	Nakahashi et al^[2]^ (2020)	Trotter et al^[16]^ (1947)
Base of triangle [Measurement Target]	80.2 ± 10.0 (58.2–109.2) [PSISs]	64.7 ± 4.7 (53–75) [SLSCs]	NA [SLSCs]	75.5 ± 10.3 [SLSCs]	60.0 ± 6.7 (20–75) [SLSCs]	NA [SLSCs]	66.5 ± 53.52 (51–79.5) [SLSCs]	NA [SLSCs]	NA [SLSCs]	NA [SLSCs]
Left border of triangle Distance between left PSIS and SA	64.1 ± 8.8 (45.6–96.7)	70.4 ± 9.6 (44–93)	NA	75 ± 10.2	60.0 ± 8.3 (37–76)	NA	67.5 ± 9.5 (46–88.1)	NA	NA	NA
Width of SH	28.6 ± 8.4 (13.8–45.2)	28.1 ± 7.1 (12–50)	25.2	21 ± 8.0	18.8 ± 7.6 (4.3–38.6)	20	32.9 ± 9.9 (12–53)	NA	20.9 ± 5.7 (6.7–39.7)	24.8
Height of SH	10.9 ± 2.7 (3.8–16.5)	13.5 ± 2.7 (9–22)	19.5	17 ± 2.6	12 ± 2.8 (6–23.3)	12.5	17.5 ± 3.2 (7–28)	10.2 ± 0.4 (2.2–18.4)	12.1 ± 2.8 (5.8–21.0)	17
Depth of SH at apex	6.3 ± 1.6 (1.6–9.5)	8.4 ± 2.0 (4–12)	5.53	4.8 ± 1.9	5.0 ± 1.6 (1.9–10.4)	4.8	4.5 ± 1.3 (1–7)	6.0 ± 1.9 (1.9–11.4)	6.0 ± 1.7 (2.4–9.7)	5.3

PSIS = posterior superior iliac spine, SLSC=superolateral sacral crest, SA=sacral hiatus apex, SH=sacral hiatus.

The width of the sacral hiatus also exhibited a wide range of values, as in previous reports. Aggarwal et al^[13]^ reported that 21% of the sacra were <10 mm, and 79% of the sacra were adequately broad for needle insertion. In our study, sacral hiatus widths <10 mm were found in thirty-six (39.5%) sacra, which were larger than those in previous reports. Sekiguchi et al^[14]^ reported that the width of the sacral hiatus was 10.2 ± 0.4 (2.2–18.4) in the Japanese population, similar to our study [10.9 ± 2.7 (range 3.8–16.5 mm)]. These results suggest that the width of the sacral hiatus in Japanese is smaller than those in other races.

The depth of the sacral hiatus at the apex needs to be large enough to insert the needle in the sacral canal. Sekiguchi et al^[15]^ stated that the diameter of the depth of the sacral hiatus <2 mm may cause CEB failure. Further, Aggarwal et al^[13]^ reported that the depth of the sacral hiatus was <3 mm in 8.7% cases. Accordingly, it may be proven difficult to insert needles. In our study, it was <2 mm in one (1.1%) sacrum, and <3 mm in another five (5.5%) sacra.

In conclusion, based on 3DCT measurements, it is inferred that the trigonum sacrale is not an equilateral triangle. This is useful information for the identification of the sacral hiatus when the landmark-based technique is used.

## Author contributions

NK and SN contributed to the conception and design of the study, manuscript writing and final review of the manuscript. YS, CI, KY and HA contributed to the conception and design of the study, and final review of the manuscript.

**Conceptualization:** Satoshi Nozawa.

**Formal analysis:** Norishige Kuramitsu.

**Supervision:** Yasumichi Sakaguchi, Kazunari Yamada, Chizuo Iwai, Haruhiko Akiyama.

**Writing – original draft:** Norishige Kuramitsu.

**Writing – review & editing:** Satoshi Nozawa.
